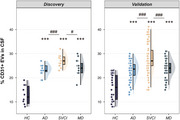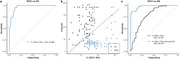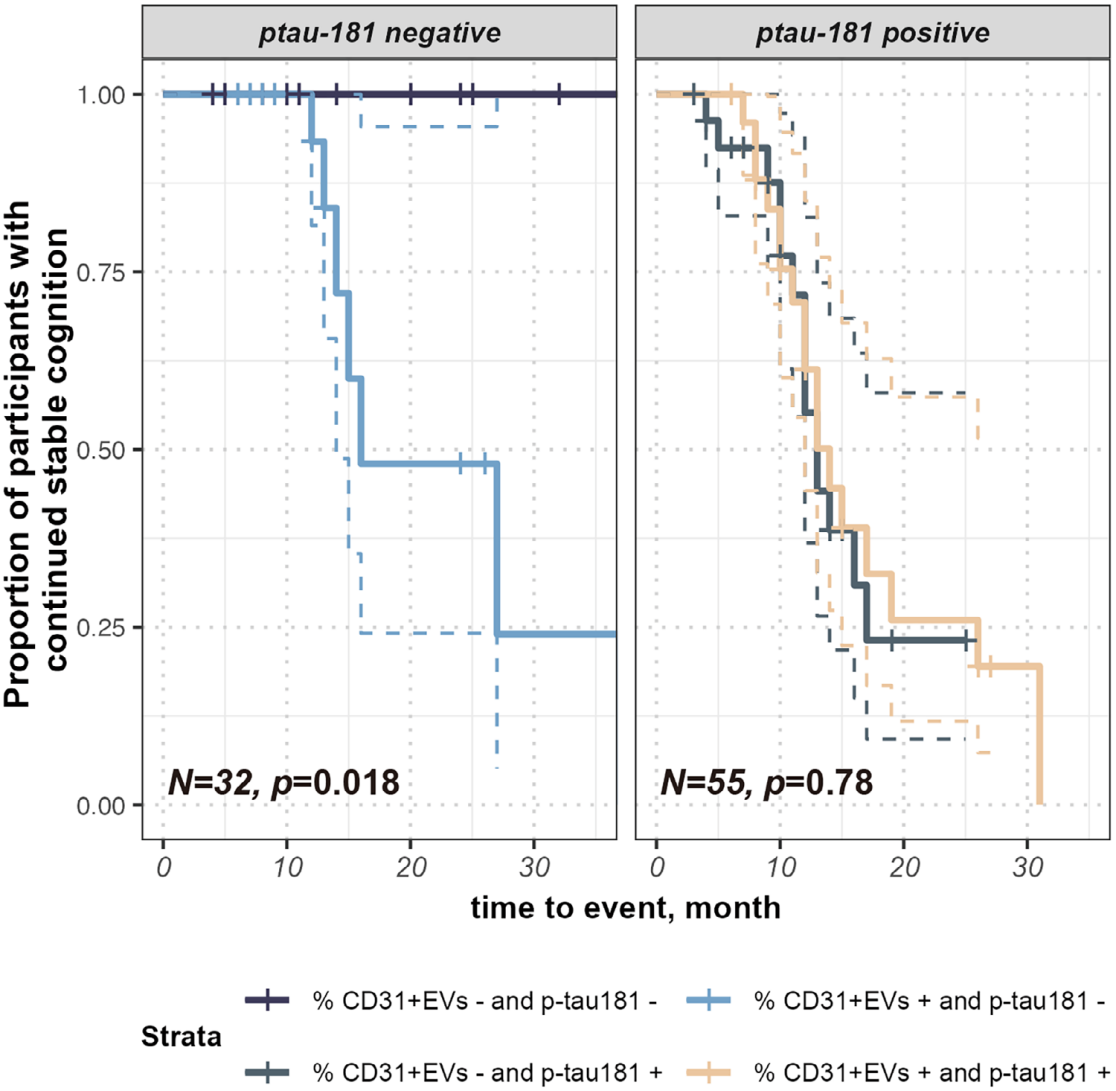# CD31^+^ small extracellular vesicles as a novel biomarker for vascular injury and cognitive impairment

**DOI:** 10.1002/alz70856_101397

**Published:** 2025-12-25

**Authors:** Tongyao You, Qi Han, Yingzhe Wang, Qiang Dong, Mei Cui

**Affiliations:** ^1^ Huashan Hospital, Fudan University, Shanghai, Shanghai, China; ^2^ MOE Frontiers Center for Brain Science, Fudan University, Shanghai, China

## Abstract

**Background:**

The 2024 framework for diagnosing and characterizing Alzheimer's disease (AD) introduces new biomarker categories including vascular brain injury (V) beyond A, T, and N. However, the diagnosis of V relies on neuroimaging, which offers lower sensitivity and specificity compared to molecular biomarkers. Our preliminary study demonstrated that cerebrovascular endothelium secretes a higher number of small extracellular vesicles (EVs) when damaged. Therefore, CD31^+^ EVs released from brain endothelial cells serve as direct indicators of vascular pathology. In this study, we conducted a hospital‐based, multicenter investigation to explore the potential of CD31^+^ small EVs as biomarkers for vascular injury.

**Method:**

A total of 346 individuals were enrolled, with 55 in the discovery cohort and 291 in the validation cohort. All participants underwent evaluations of global cognition, ATN categories, brain magnetic resonance imaging, and cerebrospinal fluid EV analysis by nano flow. They were classified into four groups: healthy control (HC), pure AD (without evident vascular imaging markers), pure subcortical vascular cognitive impairment (SVCI), and mixed dementia (MD), which includes a combination of AD and SVCI. Multivariable linear regression, receiver operating characteristic curves, and survival analysis were performed.

**Result:**

We identified an elevated percentage of CD31^+^ EVs in the SVCI group, where vascular injuries are the primary contributors to cognitive decline (Figure 1). Significant associations were observed between the percentage of CD31^+^ EVs and individual vascular risk factors (VRFs), total VRF burden, and imaging markers of cerebral small vessel disease (all *p* < 0.05). The percentage of CD31^+^ EVs demonstrated superior diagnostic accuracy in distinguishing SVCI from HC, and the combination of CD31^+^ EV and *p*‐tau181 exhibited excellent discriminative performance between SVCI and AD (Figure 2). Survival analysis revealed that, in the absence of elevated *p*‐tau181, participants with high levels of CD31^+^ EVs experienced more rapid progression of cognitive impairment (Figure 3).

**Conclusion:**

Our results highlight that the percentage of CD31^+^ EVs is significantly correlated with vascular brain injury, and the combination of CD31^+^ EVs with *p*‐tau181 shows excellent discriminative performance. This also provides an opportunity for us to identify cerebrovascular EVs from plasma, enabling early detection of changes in the vasculature.